# A Hybrid Key Management Scheme for WSNs Based on PPBR and a Tree-Based Path Key Establishment Method

**DOI:** 10.3390/s16040509

**Published:** 2016-04-09

**Authors:** Ying Zhang, Jixing Liang, Bingxin Zheng, Wei Chen

**Affiliations:** 1 College of Information Engineering, Shanghai Maritime University, Shanghai 201306, China; yingzhang@shmtu.edu.cn (Y.Z.); liangjixing501@163.com (J.L.); zhengbingxin501@163.com (B.Z.); 2 Department of Computer Science, Tennessee State University, Nashville, TN 37209, USA

**Keywords:** wireless sensor networks, key management, tree-based, path key establishment, mobile sink

## Abstract

With the development of wireless sensor networks (WSNs), in most application scenarios traditional WSNs with static sink nodes will be gradually replaced by Mobile Sinks (MSs), and the corresponding application requires a secure communication environment. Current key management researches pay less attention to the security of sensor networks with MS. This paper proposes a hybrid key management schemes based on a Polynomial Pool-based key pre-distribution and Basic Random key pre-distribution (PPBR) to be used in WSNs with MS. The scheme takes full advantages of these two kinds of methods to improve the cracking difficulty of the key system. The storage effectiveness and the network resilience can be significantly enhanced as well. The tree-based path key establishment method is introduced to effectively solve the problem of communication link connectivity. Simulation clearly shows that the proposed scheme performs better in terms of network resilience, connectivity and storage effectiveness compared to other widely used schemes.

## 1. Introduction

In most of the traditional key management schemes for wireless sensor network, the sink node is fixed, which may cause lots of data storage and forwarding among the sensor nodes, and the keys may have higher risks of being captured [1,2,3,4]. Sometimes, with the random deployment, there exist some isolated sensor nodes which cannot communicate with any sink node. Thus, many more sink nodes are usually needed to guarantee reliable data collection, which increases the system cost and energy consumption [5,6]. Mobile Sink (MS) nodes with abundant resources can move within the range of the whole network, which not only reduces the amount of data storage and forwarding, but also decreases the energy consumption and network communication overhead, and meanwhile, it can effectively avoid the appearance of isolated nodes [7,8,9,10]. There are many constraints in wireless sensor networks because of the lack of energy resources, limited communication range, low transmission power and poor computing abilities [11,12,13,14,15,16]. Thus, sometimes nodes cannot use an asymmetric key encryption mechanism during communications, or they cannot get the geographic information after node deployment, or the storage capacity to store more information is limited, and so on. MS nodes will persistently broadcast their own identities on the move, and sensor node within communication range will send data stored in itself to the MS node after receiving the handshaking messages. In case the nodes are captured by an adversary, the adversary can acquire the information transmitted in the network by attacks including forgery, modification, and replay. They can prevent the MS node from receiving date from the sensor nodes, or even reduce the lifetime of the network as well.

Existing key management schemes proposed for WSNs cannot solve the problems mentioned above well. First, most schemes are designed for networks with fixed sink nodes, which is not applicable for future application environments. Secondly, the generation and establishment of the keys mostly depend on a single encryption method, so the keys are easily captured and identified, which leads to lower network security. In addition, research on path key establishment and maintenance based on multi-hop links is insufficient for the current key management schemes. Thus in most cases, when the system security is improved, at the same time the connectivity of the network will be decreased.

This paper proposes a hybrid key management scheme (PPBR scheme) based on a polynomial pool-based key pre-distribution and basic random key pre-distribution. The scheme combines the advantages of the two protocols, utilizes the *t*-degree property of polynomials and improves the security of the traditional basic random key pre-distribution scheme. It makes the adversary need to capture a large number of nodes in the network to decode the keys, since it has to possess the polynomial coefficients and random keys at the same time in order to capture the uncompromised nodes. Therefore, the scheme improves the security of the network and enhances the network's ability to resist capture attacks. Furthermore, the proposed scheme puts forward a path key generation method based on the tree-based path key establishment, which regards the MS node as the root in the range of communication. This can deal with the problem of higher nodes’ storage capacity requirements and poor network connectivity. Simulation and analysis prove that the proposed method has better storage efficiency, connectivity and resilience for WSNs with MS compared to other widely used key management schemes.

The rest of this article is organized as follows: in Section 2, some background knowledge and related work on key management schemes are introduced. Then, in Section 3, we describe the PPBR key management scheme in detail. Section 4 presents the simulation and results analysis, and finally we conclude the article in Section 5.

## 2. Related Works

Eschenauer and Ghgor were the first to present a key management scheme based on random probability (the so-called basic random key distribution scheme, or E-G scheme [17]) for WSNs, which is the foundation of the other key management schemes. The scheme randomly deposits partial keys on the basis of pre-setting all pairwise keys, so it can greatly decrease the node resources cost on the premise of maintaining a certain connectivity in the network. The basic random key pre-distribution scheme concept can be roughly summed up as the following process:

(1) Key pre-distribution. The server (usually in the base station) creates a big key pool *M* and each key has a unique *ID* identifier. Nodes pre-store *K* keys randomly selected from the pool and build the key ring. This ensures that two nodes can share at least one of the keys at a certain probability.
(2) Shared-key discovery. Each node gets the shared-key by matching the key ring in its own storage with identifier broadcasting.
(3) Path key establishment. If the two nodes do not have the shared-key directly, they can develop the path key through the intermediate nodes. The disadvantages of this scheme are obvious, such as the utilization of keys in the key ring is lower, the same key will be established by different nodes, and it will reduce robustness of the system.

Chan *et al.* [18] proposed an improved random key distribution scheme, called *q*-composite scheme, which increases exponentially the difficulty for an adversary to destroy the safety link, but it reduces the network connectivity. Choi *et al.* [19] developed a new robust key predistribution scheme by using keys assigned based on the notion of eigenvalues and eigenvectors of a square matrix of a keys pool. Zhou *et al.* [20] proposed a key predistribution scheme combining the Chinese Remainder Theorem (CRT) with a LU matrix. The scheme achieves smaller storage overhead and better network resilience. Such schemes can be classified as key pre-allocation schemes based on key pools. They use the same key to establish a session key between the nodes, and it can increase the connectivity when the nodes store a certain number of keys, but the network security is usually not better.

Liu *et al.* proposed a modified scheme in [21] based on the Blundo *et al.* scheme [22], namely a key predistribution scheme based on polynomial pool, and two possible instantiation schemes were presented. In this scheme, the server randomly generates *S* bivariate *t*-degree polynomials: {*f_i_*(*x,y*)}, *i* = 1,2,…S:

(1) f(x,y)=∑i,j=0taijxiyj=at0xt+ax(t−1)(t−1)1y+⋅⋅⋅+a1(t−1)xy(t−1)+a0tyt

The polynomials have the property *f*(*x,y*) = *f*(*y,x*) and each *a_ij_* is different and completely confidential for each node. Prior to the deployment, each node randomly selects *m* polynomials, where, 1 ≤ *m* ≤ *S*, and shares polynomials at a certain probability. After nodes are deployed, if two nodes find there exist shared polynomials, they can calculate the direct session key by exchanging the binomial *ID* identifiers and putting the *ID* into the binomial, otherwise the two nodes establish a session key with path key agreement. In this scenario, the *t*-degree polynomial has a safety threshold *t* (*t*-degree property), and the key information in other nodes will not be influenced by the captured nodes as long as the number of captured nodes is less than *t*. The scheme needs to calculate the value of the polynomial during the key establishment, so the computation cost will be increased. However, it will be able to get in return security for the whole network as long as the computational overhead requirement of sensor nodes is satisfied.

With increasing network scale, the number of nodes probably captured by an adversary will increase, which makes the polynomial lose its *t*-degree property easily. The safety threshold can be improved by increasing the degree of the polynomial, but it means that the node’s storage and computational overhead will be significantly higher for the limited resources of sensor nodes. Besides the current ideas for establishing a shared key, Wang *et al.* [23] presented the multiple asymmetric quadratic form of a polynomial, which generates the session key by the relationship between eigenvalues and eigenvectors of the quadratic form. The modified Liu scheme [24] increases the rate of direct connection by predistributed polynomials in a heterogeneous network. In recent years, many scholars have put forward some combined methods with different predistribution schemes. The Amar scheme [25] combines the polynomial pool-based key pre-distribution with the probabilistic generation key pre-distribution scheme [26]. In fact, the Amar scheme assigns the same number of polynomials and keys in MS and sensor nodes, and it has not fully made use of the heterogeneity of the networks to enhance the security performance of the system. In addition, the Amar scheme establishes communication links only based on the probability, and its connectivity is relatively lower. Huang’s scheme [27] builds the key pairs based on the LEACH protocol, which generates the key pool and ternary polynomial. These kinds of schemes can be classified as key predistribution scheme based on polynomials, and their objective is to establish a unique session key for any two nodes under the condition of having the same polynomial. However, polynomials’ *t*-degree property makes the scale of the network limited, and its connectivity and security cannot be guaranteed by the limited resources. Tree-routing generation protocols for wireless sensor networks were proposed in [28,29,30], and they have been used in routing selection, relay configuration and probabilistic top-k queries, but they were only used in static networks.

In this paper, the PPBR scheme assigns different number of polynomials and keys in MS and sensor nodes to make the polynomial ring, and the heterogeneity of PPBR will improve its security performance compared to the homogeneity of the Amar scheme. In addition, compared to the method of communication link establishment only based on probability in the Amar scheme, the tree-based path key establishment method in the PPBR scheme can improve the connectivity probability of establishing communication links. We use the tree-based method to establish the path key for key management. It can establish a dynamic indirect communication link between the MS and the sensor nodes who cannot communicate with the MS directly. The dynamic tree-based path key establishment can improve the key connectivity for key management of the network.

## 3. The PPBR Key Management Scheme

### 3.1. Network Model and Hypothesis

**Figure 1:**
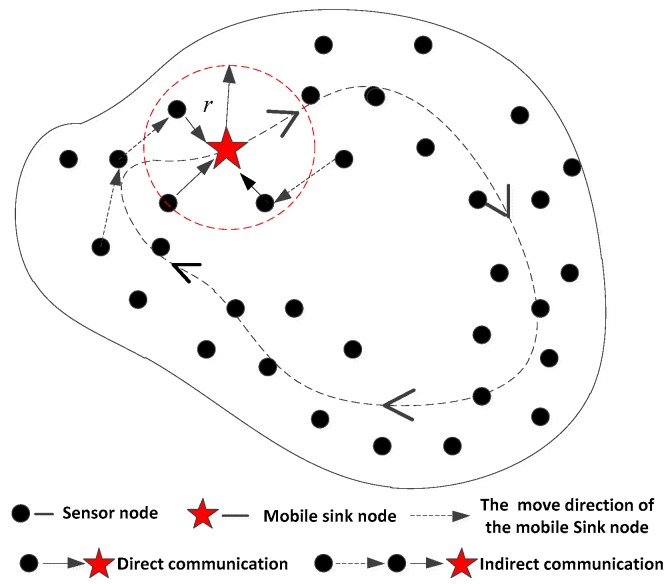
A schematic diagram of a wireless sensor network with a mobile sink.

This article assumes that sensor nodes can temporarily store the sensed data, all the data is managed by the network server in the base station, and MS node is dispatched to retrieve and collect the data regularly. In marine environment monitoring, a surveillance ship can be regarded as a MS which can converge the monitoring data. In battlefield reconnaissance, a mobile communication vehicle can also be regarded as a MS which can collect the battlefield information from the nodes of sensor networks in the future informatization war. The MS contains the sole *ID* identifier of the whole network, and it has more abundant computation, storage, and energy resources than ordinary sensor nodes. However, the traditional scheme with fixed sink nodes, usually requires a large number of sink nodes distributed in the network. In addition, the heterogeneity on storage space, energy and computational ability between fixed sink nodes and the ordinary sensor nodes is not so very different from the heterogeneity between a MS node and ordinary sensor nodes. A schematic diagram of a wireless sensor network with MS is shown in Figure 1.

There are mainly three kinds of moving patterns for sink nodes, which includes random route movement [31], fixed route movement [32], and controlled route movement [33,34]. In this article, the sink node moves among fixed sensor nodes, and collects the monitoring data uploaded by the ordinary sensor nodes within their communication range, and the sensor nodes which are out of the communication range will be in the sleep mode. In fact, when the MS broadcasts the information within its communication range, it does not need to know whether the sensor node *S_i_* is its neighbor node or not. Many sensor nodes in the network are limited in storage space, communication distance and energy supplies, and they will remain in a static state after deployment. Nevertheless, the MS node has relatively more abundant resources and is equipped with tamper-proof hardware and safety detection equipment, so it is reasonable to assume that generally a MS node would not be captured. This kind of network system can implement more complex data fusion, data access, data transmission, data forwarding and routing service by using a MS with its abundant resources. This manner can greatly reduce the communication overhead, and energy consumption in the ordinary nodes and effectively avoid generating isolated nodes in the network.

**Figure 2:**
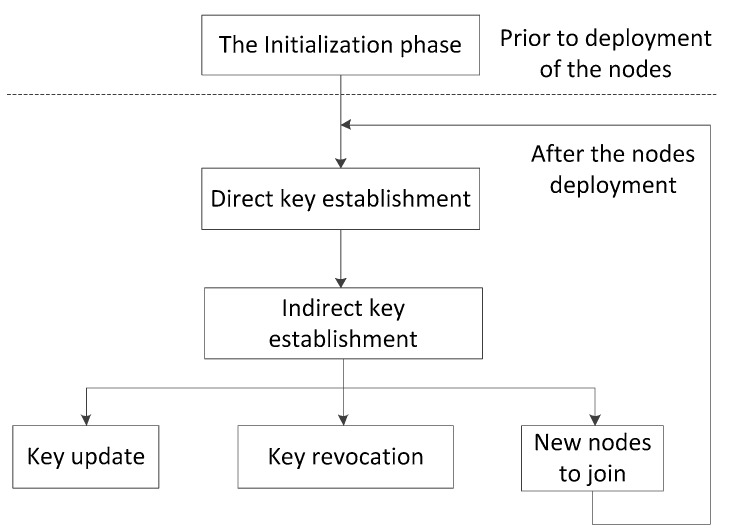
The main process of the PPBR scheme.

**Figure 3:**
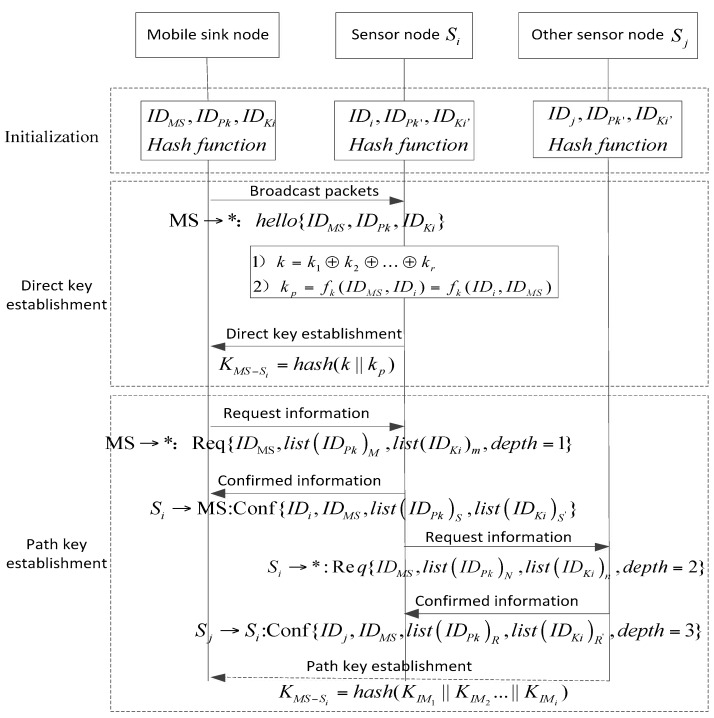
The steps of key establishment for the PPBR scheme.

The proposed PPBR scheme includes the initialization phase, direct key establishment, path key establishment, the key revocation, update, and the joining in of new nodes. The process of the scheme is shown in Figure 2, and the main steps of key establishment are shown in Figure 3.

The proposed PPBR key management scheme is a combination of the Polynomial Pool-based key predistribution and Basic Random key predistribution scheme. The scheme combines the advantages of the two protocols, utilizes the *t*-degree property of polynomial to improve the security of the traditional random key predistribution scheme. In detail, we use the Polynomial Pool-based key predistribution scheme to create the polynomial pool F which includes *S_p_* *t*-degree bivariate polynomials and use the Basic Random key pre-distribution scheme to create the key pool K which contains *S_k_* keys. Regardless of the establishments of direct session keys or path keys, they are all need to be calculated by the hash function using shared keys and shared polynomials. It makes the adversary need to capture a large number of nodes in the network to decode the keys, since it has to compromise the polynomial coefficients and random keys at the same time in order to capture the uncompromised nodes.

### 3.2. The Initialization Phase

The server generates polynomial pool *F* including Sp *t*-degree bivariate polynomials, the polynomial can be expressed as Equation (2):

(2) $$f_{k}(x,y)=\sum_{i,j=0}^{t}a_{ij}x^{i}y^{j}$$

where k=1,2,⋅⋅⋅,Sp, and it satisfies *f*(*x,y*) = *f*(*y,x*). 

Each polynomial contains a unique identifier *ID_Pk_* (*k* = 1,2,…,*S_p_*), and it creates the key pool *K* which contains *S_k_* keys. Each key has a unique identifier *ID_Ki _*(*k* = 1,2,…,*S_k_*) as well. Before deployment, the MS node needs to pre-store the following key information: the identifier *ID_MS_*, the polynomial ring which consists of *M* polynomials selected from the polynomials pool *F*, and the key ring which consists of *m* keys selected from the keys pool *K* randomly by the server. On the other hand, sensor nodes need to pre-load the identifier *ID_i_*, the polynomial ring which consists of *N* polynomials selected from the polynomials pool *F*, and the key ring which consists of *n* keys randomly selected from the keys pool *K* by the server. The MS and sensor nodes both need to store the hash function *H*.

### 3.3. Direct Key Establishment Stage

The sink node broadcasts packets within its communication range while it moves along a fixed path. The information contains its own *ID* identifier, the polynomial ring and the key ring: MS →*: hello{*ID_MS_,ID_Pk_,ID_ki_*}. Sensor node *S_i_* in the communication range matches the received packets with their own information. If *ID_Pk_* of the polynomial ring and *ID_Ki_* of key ring can both be matched, the *S_i_* will determine to share the keys with MS. Once MS and *S_i_* at least have one shared polynomial and one shared key, they can establish the session key directly by the following process:

(1) If there are *r* shared keys in the key ring, then *k* = *k*_1_⊕ *k*_2_⊕…⊕ *k_r_*.
(2) If there are *R* shared polynomials in the polynomial ring, then chooses a polynomial *f_k_*_(_*x,y*) randomly and calculates: *k_p_* = *f_k_*(*ID_MS_*,*ID_i_*) = *f_k_*(*ID_i_,ID_MS_*).
(3) Finally, the direct session key between MS and sensor nodes can be calculated by the function *H*: *K_MS–Si_* = *hash*(*k*‖*k_p_*).

In the session key transmission process, we can take the secure transmission method based on the ECC with public-private encryption. The ordinary nodes encrypt the shared key with the public key and the MS decrypts it with the private key, which can ensure the key cannot be decrypted in case the key is intercepted in the transmission process.

### 3.4. Path Key Establishment

**Figure 4:**
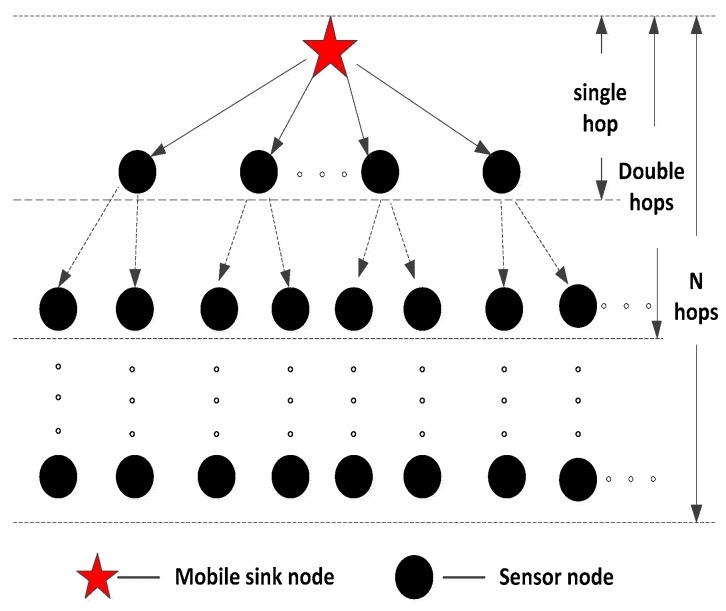
Path key establishment method via the tree-based construction.

PPBR scheme establishes the communication link based on a certain probability, thus there are some nodes which may not be able to communicate with the MS node, and this will reduce the network connectivity. It must establish an indirect communication link in the path key establishment phase when the MS and sensor nodes cannot establish a session key directly. The path key establishment method used in this article is different from the conventional polynomial key management scheme, and it utilizes the tree-based construction method in wireless sensor networks [35]. It builds a tree which regards the sink node as a root within the scope of the sink node’s communication. The schematic diagram is shown as Figure 4.

Suppose that the *status* and the *depth* denote whether the nodes are in the range of sink node’s communication and the depth of the tree, respectively. The initial values of *status* and *depth* are all 0. The steps of the process can be summarized as follows:

First, MS broadcasts request information to sensor nodes within the scope of communication, the information contains MS identifier, *ID* table (*ID* identifiers of *M* polynomials) of the polynomial ring, and *ID* table (*ID* identifiers of *m* keys) of the key ring: MS →*: Req{*ID_MS_,list*(ID*_Pk_*)*_M_,list(ID_Ki_*)*_m_*,depth = 1}.

Second, sensor node *S_i_* within the range of communication will set the value of *status* as 1, and then match the *list*(ID*_Pk_*) and *list(ID_Ki_*) received from the MS with the polynomials and keys stored inside the sensor node. If there are shared polynomials and the keys, the sensor node *S_i_* makes the value of *depth* add 1. Then, the sensor node *S_i_* will send response packets to the MS, the information contains: sensor nodes *ID*, mobile node *ID*, the shared polynomial *ID* table (*ID* identifiers of *S* polynomials, 1 ≤ *S* ≤ *N*), and the shared key *ID* table (*ID* identifiers of *S*’ keys, 1 ≤ *S’* ≤ *n*): *S_i_* → *MS*: Res{*ID_i_,ID_MS_,list*(ID*_Pk_*)*_S_,list(ID_Ki_*)*_S’_*}.

If there are no shared polynomials and the shared keys, the sensor node *S_i_* will discard the broadcast packets from the MS.

Finally, sensor node *S_i_* broadcasts request information to its neighbor nodes again, the information includes: MS identifier *ID_MS_*, *ID* table (*ID* identifiers of *N* polynomials) of the polynomial ring, and *ID* table (*ID* identifiers of *n* keys) of the key ring which are all pre-stored in the sensor node: *S_i_* → *: Req{*ID_MS_,list*(ID*_Pk_*)*_N_,list(ID_Ki_*)*_n_*,*depth* = 2}.

In the scope of the node’s communication, the sensor node *S_i_* will make the value of *depth* add 1, and send the response information at the same time once finding out the shared polynomials and keys.

With the above steps, sensor nodes within the range of communication can be joined into the tree as much as possible, so that sensor nodes are able to increase connectivity by establishing a multi-hop communication link to the sink node. The session key between target node MS and source node *S_i_* can be obtained by the indirect session key *K_IMi_* established by the intermediate nodes: *K_MS–Si_* = *hash*(*K_IM_*_1_‖*K_IM_*_2_…‖*K_IMi_*).

### 3.5. Key Updating and Revocation

When the residual energy of sensor nodes is less than a certain threshold, they will automatically report to the MS node, and send disengaging requests automatically. The MS removes the disengaging node’s information from the *ID* table, and deletes all key information associated with the departing node after sending the MS reply confirmation. When sensor nodes are captured by an adversary, the network will find the compromised nodes by using the intrusion detection mechanism, and then the MS will delete all the information related to the captured nodes. 

After the MS sets up a session key with sensor nodes, it can communicate with sensor nodes safely with the session key by using a symmetric encryption algorithm. Symmetric encryption algorithms have the advantage of lower energy consumption, but the session key is easy to crack when the same session key is used for a long time, so the session key should be updated regularly. The MS launches a key update at interval of time *T*, generates a random number *r* which is encrypted by session key: message = *E_KMS–Si_*(*r*), and then broadcasts the message to the sensor nodes which already had established a session key with the MS. Sensor nodes will receive the message, decrypt the random number *r* with the corresponding session key, and meanwhile they will complete the key update with the *H* function: *K’_MS–Si_* = *hash*(*K_MS–Si_* ‖*r*). After the updating, the random number *r* will be deleted.

### 3.6. Joining Into of the New Nodes

When a new node *S_i_* joins the network, the unique identifier $ID_{i}$, *N* polynomials and *n* keys which are randomly selected and assigned from the polynomials pool *F* and the keys pool *K* by the server respectively, and the hash function *H* will all be pre-loaded. When the new node is within the communication radius of the MS node, the MS will launch the identity authentication for the new node. After confirming the node’s legality, it will establish a session key by using the method mentioned above.

## 4. Simulations and Analysis

### 4.1. Storage Effectiveness Analysis

The proposed scheme assigns different number of polynomials and keys in the MS node and sensor nodes, and this reflects the heterogeneity property between the two kinds of nodes. Compared to homogeneous network, for example, Amar scheme assigns the same number of polynomials and keys to MS and sensor nodes. In the case of achieving the same connectivity, the proposed scheme can significantly reduce the storage overhead. The probability *P*1 of sharing at least one polynomial between MS and sensor node $S_{i}$ can be expressed as Equation (3):

(3) $$\begin{array}{ll} P1 & =1-P(do\;not\;share\;any\;polynomial\;between\;MS\;and\;S_{i})\\ & =1-\frac{\left(\begin{array}{c} Sp\\ M \end{array}\right)\left(\begin{array}{c} Sp-M\\ N \end{array}\right)}{\left(\begin{array}{c} Sp\\ M \end{array}\right)\left(\begin{array}{c} Sp\\ N \end{array}\right)} \end{array}$$

where *S_p_* is the size of polynomial pool, *M* is the size of polynomial ring in MS, and *N* is size of the polynomial ring in *S_i_*.

The probability *P*2 of sharing at least one polynomial between MS and sensor node *S_i_* in homogeneous network can be expressed as Equation (4):

(4) $$\begin{array}{ll} P2 & =1-P(do\;not\;share\;any\;polynomial\;between\;MS\;and\;S_{i})\\ & =1-\frac{\left(\begin{array}{c} Sp\\ S \end{array}\right)\left(\begin{array}{c} Sp-S\\ S \end{array}\right)}{\left(\begin{array}{c} Sp\\ S \end{array}\right)\left(\begin{array}{c} Sp\\ S \end{array}\right)} \end{array}$$

where *S_p_* is the size of polynomial pool, and *S* is the size of polynomial ring in the nodes (including MS and *S_i_*). In this case, MS and sensor nodes store the same size of polynomial ring.

**Figure 5:**
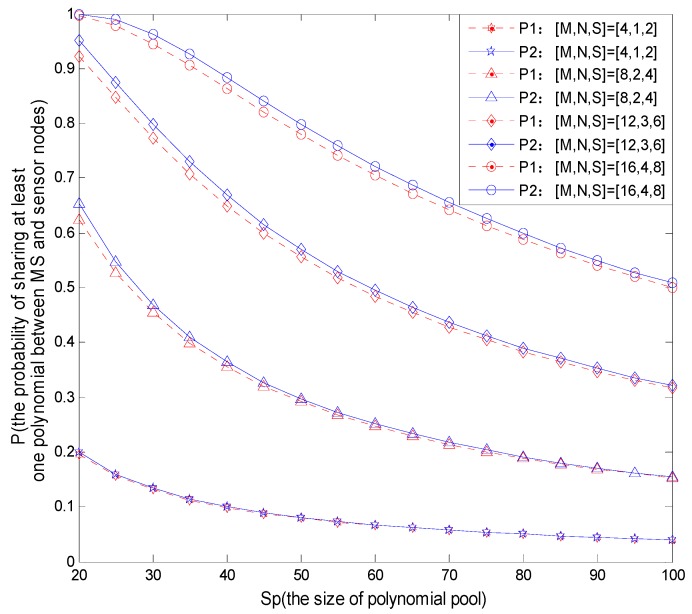
The probability of sharing polynomial with different *S_p_*.

For the two different structures of the network, the different values of *M*, *N*, *S* will have different influences on storage overhead, because the more polynomials are stored in a node, the larger the storage space cost will be. We can get the simulation results as shown in Figure 5.

This article takes an example of polynomial distribution, and the key distribution method follows the same way. In Figure 5, the parameters set [*M*, *N*, *S*] in four curves from bottom to up are taken the values as: [4, 1, 2], [8, 2, 4], [12, 3, 6] and [16, 4, 8] respectively, where *M*, *N* and *S* satisfy the equation: *M* *×* *N* = *S*^2^. According to Figure 5, on the premise of ensuring the same sharing probability, we can adjust the values of *M* and *N* properly to minimize the number of polynomials which are stored in the sensor nodes, and let the MS with more resources store more polynomials. For example, when [*M*, *N*, *S*] = [16, 4, 8] and the size of polynomial pool *S_p_* = 60, the probabilities to share at least one polynomial for homogeneous and heterogeneous networks are: *P*1 = 0.71 and *P*2 = 0.7, respectively. 

At this time, although the two probabilities are nearly similar, the number of polynomials stored by sensor nodes in a homogeneous network is: *S* = 8, and the number for a heterogeneous network is: *N* = 4. It indicates that the proposed scheme can save sensor node storage space, and it improves the storage efficiency by using different disposal schemes for the storage between MS nodes and fixed sensor nodes. 

The existence of MS nodes makes the fixed sensor nodes’ communication no longer rely too much on cluster heads. When MS nodes do not communicate with the sensor nodes, the sensor nodes will not communicate with each other, and they will remain in sleeping mode, thus reducing the communication overhead among the sensor nodes. However, due to the adoption of the polynomial method, the protocol will increase the computation overhead relatively in the process of key establishment for sensor nodes. In order to improve the robustness of the whole network, it is worthy contributing some computation overhead.

### 4.2. Connectivity

#### 4.2.1. Directly Establishing Communication Links

The probability *q* of sharing at least one key between MS and sensor node *S_i_* can be expressed as Equation (5):

(5) $$\begin{array}{ll} q & =1-Q(do\;not\;share\;any\;key\;between\;MS\;and\;S_{i})\\ & =1-\frac{\left(\begin{array}{c} S_{k}\\ m \end{array}\right)\left(\begin{array}{c} S_{k}-m\\ n \end{array}\right)}{\left(\begin{array}{c} S_{k}\\ m \end{array}\right)\left(\begin{array}{c} S_{k}\\ n \end{array}\right)} \end{array}$$

where *S_k_* is the size of the key pool, *m* is the size of key ring stored in MS, and *n* is the key ring size of sensor node *S_i_*. Therefore, the probability *p* of MS establishing a direct communication link with sensor node *S_i_* can be represented as *p* = *P*1 × *q*. In the Amar scheme, MS and sensor node *S_i_* select the same number of polynomial to build the polynomial ring, so the probability *p*’ of the scheme establishing a direct communication link can be represented as *p’* = *P*2 × *q*.

**Figure 6:**
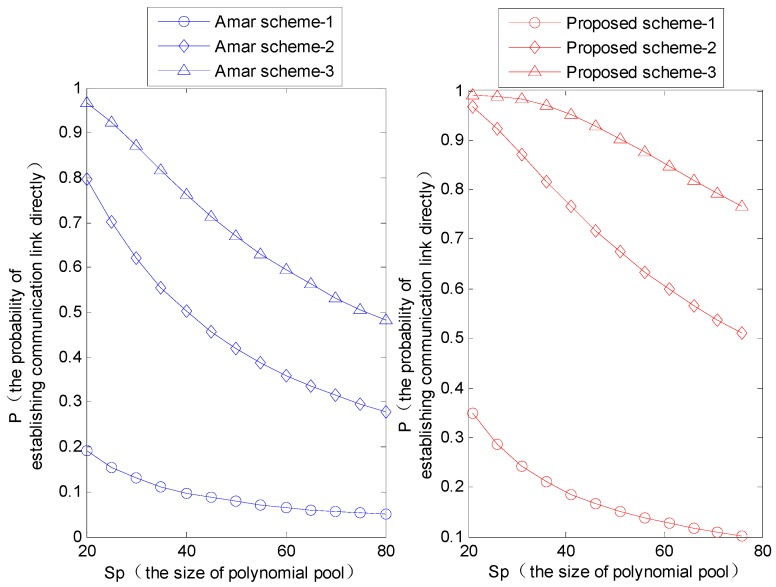
The probability that MS and sensor nodes directly establish the communication link in different size of polynomial ring and polynomial pool when *q* = 0.99.

In Figure 6, the parameters in the three curves from bottom to top take the values in sequence as follows:

Amar scheme:
  Amar scheme-1: *n* = 2, *S_k_* = 111, *m* = 100, *S* = 2.
  Amar scheme-2: *n* = 5, *S_k_* = 168, *m* = 100, *S* = 5.
  Amar scheme-3: *n* = 10, *S_k_* =275, *m* =100, *S* = 7.
The proposed scheme:
  Proposed scheme-1: *n* = 2, *S_k_* = 111, *m* = 100, *N* = 2, *M* = 4.
  Proposed scheme-2: *n* = 5, *S_k_* = 168, *m* = 100, *N* = 5, *M* = 10.
  Proposed scheme-3: *n* = 10, *S_k_* = 275, *m* = 100, *N* = 7, *M* = 14.

As shown in Figure 6, the proposed scheme which selects a different number of polynomials to store in the sink node and sensor nodes can achieve a higher probability of establishing direct communication links than the Amar scheme which selects the same number of polynomials to store in these different nodes. 

**Figure 7:**
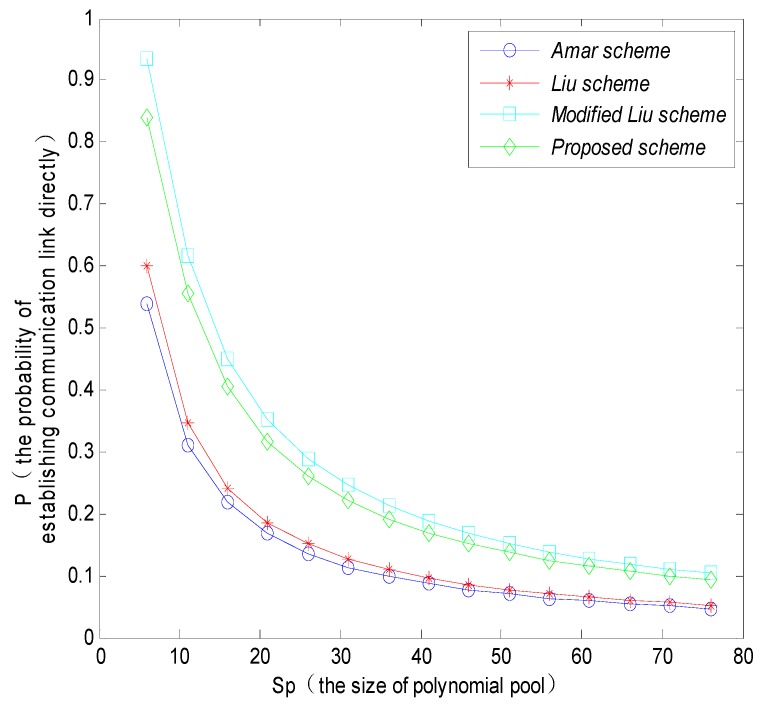
The probability that MS and sensor nodes directly establish the communication link with different schemes.

Meanwhile, the probability that the MS directly establishes a session key with sensor nodes could increase with the increasing number of polynomials and keys stored in sensor nodes, and could decrease with the increasing of the size of polynomial pool. Figure 7 shows that the directly connected probability of the proposed scheme is higher than that of the Amar scheme and Liu scheme, but it is a little bit lower than the modified Liu scheme. The reason is that the modified Liu scheme only considers assigning a different number of polynomials to mobile nodes and fixed sensor nodes, while the proposed scheme also takes into account the probability of using the shared keys simultaneously to improve the robustness of the network in the process of calculating the direct connectivity.

#### 4.2.2. Establishing a Communication Link with Multi-Hops

The method discussed above will reduce the connectivity of the network to some extent. In order to improve the connectivity, we introduce a tree-based path key establishment method to make as many nodes as possible connect to the tree with the sink node. It defines *p*(*n*) as the probability that any sensor node *S_i_* establishes a communication link with MS within *n* hops, so *p*(*n –* 1) – *p*(*n –* 2) will signify the probability that sensor nodes can establish communication link with MS within *n*-1 hops. If we define the probability that any two sensor nodes *S_i_* and *S_j_* can directly establish a communication link as *p_ss_,* thus the probability of establishing communication link with MS in only *n* hops is *p_ss_ ×* (*p*(*n –* 1) – *p*(*n –* 2)). Suppose that there are *d* sensor nodes in the communication range of the sink node, then the probability that sensor nodes cannot establish a communication link with the MS in *n* hops is (1 – *p_ss_ ×* (*p*(*n –* 1) – *p*(*n –* 2)))*^d^*. The probability that nodes cannot establish a communication link within *n* − 1 hops is 1 – *p*(*n –* 1). Thus the probability of failing to establish communication link within *n* hops can be expressed as Equation (6):

(6) $$(1-p(n-1))\cdot {\left(1-p_{ss}\cdot (p(n-1)-p(n-2))\right)}^{d}$$

The probability that a sensor node can establish a communication link with MS within *n* hops can be derived as Equation (7):

(7) $$p(n)=1-(1-p(n-1))\cdot {\left(1-p_{ss}\cdot (p(n-1)-p(n-2))\right)}^{d}$$

For simulation convenience, this article discusses the establishment of a communication link within two hops, so Equation (7) can be simplified as Equation (8):

(8) $$p_{d}=1-(1-p)\cdot {\left(1-p_{ss}\cdot p\right)}^{d}$$

where, $p_{ss}=(1-\frac{\left(\begin{array}{c} S_{p}-N\\ N \end{array}\right)}{\left(\begin{array}{c} S_{p}\\ N \end{array}\right)})\cdot (1-\frac{\left(\begin{array}{c} S_{k}-n\\ n \end{array}\right)}{\left(\begin{array}{c} S_{k}\\ n \end{array}\right)})$, *S_p_* and *S_k_* and denote the size of polynomials pool and keys pool respectively, *N* and *n* are the size of polynomial ring and key ring stored in the sensor nodes, respectively.

**Figure 8:**
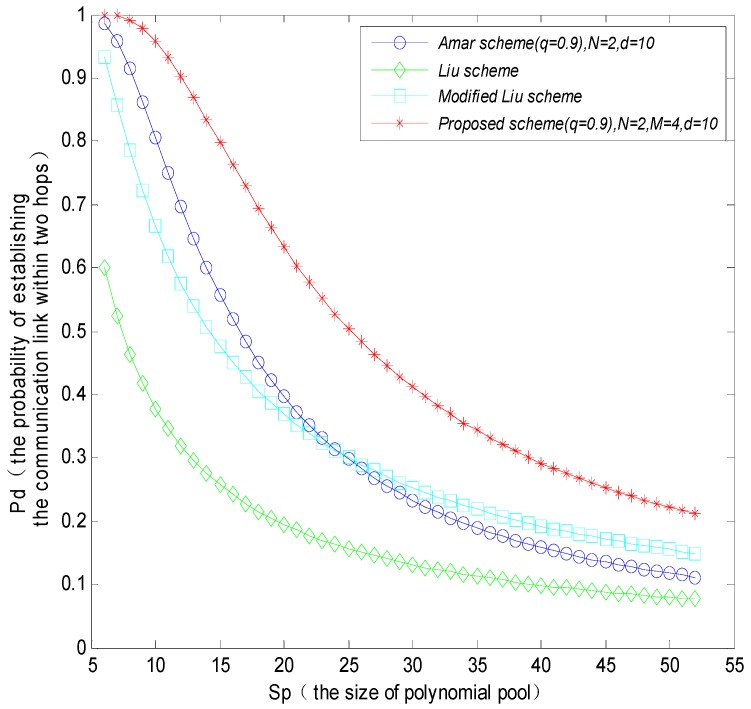
The probability that a MS and sensor nodes establish a communication link within two hops when the number of neighbor nodes *d* = 10.

**Figure 9:**
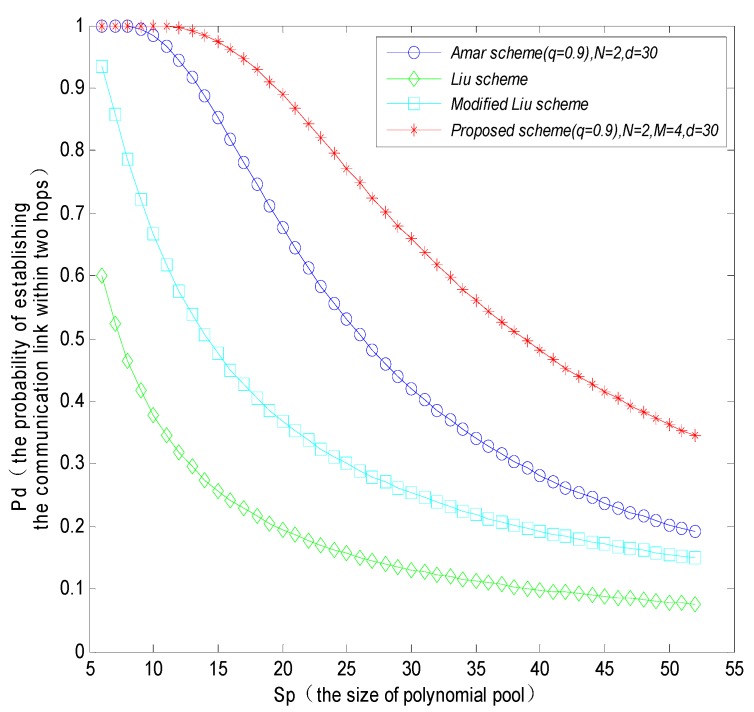
The probability that a MS and sensor nodes establish a communication link within two hops when the number of neighbor nodes *d* = 30.

In Figure 8, when the number of neighbor nodes *d* = 10, the probability of establishing a communications link within two hops between sensor nodes and MS will reduce with the increasing of the size of polynomial pool, and the probability of establishing a communications link within two hops for the proposed scheme will be higher than that in the Amar scheme, Liu scheme and the modified Liu scheme, respectively. Comparing Figure 8 and Figure 9, the probability of establishing a communication link within two hops will increase with the increasing number of neighbor nodes within the communication range of a MS. As analyzed from the simulation result, the proposed tree-based path key establishment method improves the probability of establishing a communication link significantly. The reason is that it forms a dendroid local network structure within the communication range of mobile nodes. The tree-based structure allows the sensor nodes which cannot directly establish session keys with the MS to more easily establish session key with the MS via the intermediate nodes by multi-hops, whereas the other schemes establish communication links only based on the probability, which cannot better solve the problem of lower connectivity probability. 

### 4.3. Resilience

Resilience is an important safety performance index in a key management scheme. It indicates the probability of the exposure of the session keys among the remaining uncompromised nodes after some of the nodes are captured. The proposed key management scheme forms a polynomial pool with the key pool. It makes full use of the threshold character in the polynomial key scheme, and it makes the adversary have to crack both the polynomial coefficients and the shared keys simultaneously after a large number of nodes are captured, before it can influence the other uncompromised nodes. This greatly enhances the robustness of the entire network. Normally the MS is safe, but a large number of fixed sensor nodes have the risk of being captured. If there are *x* captured nodes, the probability *p_k_* that the key in the key ring could be captured in any pair of uncompromised nodes can be expressed as Equation (9):

(9) $$p_{k}=1-{\left(1-\frac{n}{S_{k}}\right)}^{x}$$

Suppose that the number of captured nodes: *x* > *t* (where *t* denotes the degree of the polynomial). Only if the number of captured nodes is greater than the degree of the polynomial, the adversary could have the possibility of cracking the polynomial coefficients. The probability that any polynomial *f_k_*(*x,y*) is randomly selected by the sensor nodes is *n*/*S_p_*, and the probability *p_j_* that the polynomial is contained by *j* nodes in the *x* captured nodes can be expressed as Equation (10):

(10) $$p_{j}=\left(\begin{array}{c} x\\ j \end{array}\right){\left(\frac{n}{Sp}\right)}^{j}{\left(1-\frac{n}{Sp}\right)}^{x-j}$$

Therefore, the probability *p_p_* of polynomial exposure in the uncompromised sensor nodes can be expressed as Equation (11):

(11) $$p_{p}=1-\sum_{j=0}^{t}p(j)$$

According to Equations (9) and (11), the probability *p_link_* that the safety link is captured can be expressed as Equation (12):

(12) $$p_{link}=p_{k}\cdot p_{p}$$

The simulation parameters are set as follows:

(1) *P*1 = 0.5055 (*N* = 2, *M* = 4, Sp = 14, *t* = 100), *q* = 0.99 (*n* = 5, *S_k_* = 1000, *m* = 600);
(2) *P*2 = 0.3335 (*N* = 2, *M* = 4, Sp = 22, *t* = 100), *q* = 0.99 (*n* = 5, *S_k_* = 1000, *m* = 600).

**Figure 10:**
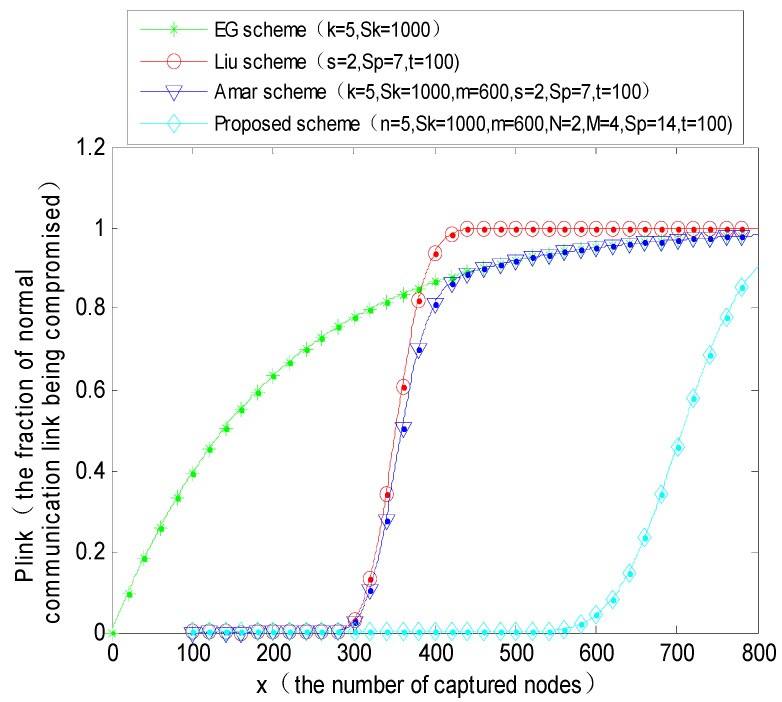
The probability that a normal communication link is captured when the direct connectivity *p* = 0.5.

**Figure 11:**
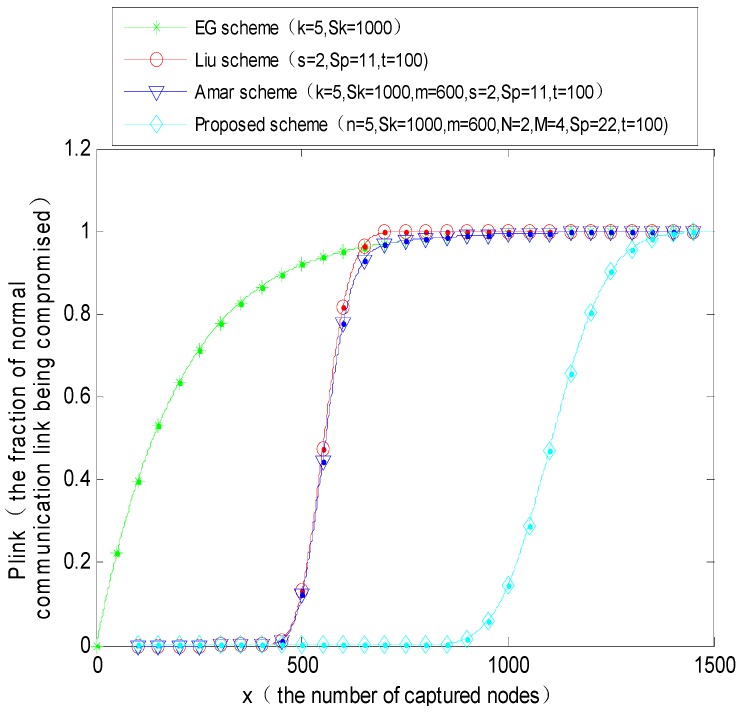
The probability that a normal communication link is captured when the direct connectivity *p* = 0.33.

From the above simulation results, we can draw the conclusion that the resilience performance of the proposed scheme is better than that of the basic random predistribution scheme (E-G scheme), polynomial predistribution scheme (Liu scheme), and Amar scheme, respectively. When the capture probability of a normal communication link is a certain value, the number of permitted captured nodes is far greater than the number of the other three schemes. For example, when the direct connectivity *p* = 0.33 and the number of captured nodes in the network reaches 650, the probability that a normal communication link is captured is as high as about 0.95 in these three schemes (EG scheme, Liu scheme and Amar scheme), in this case, the network is in dangerous status, but for the proposed scheme, for the capture probability to reach 0.95, the adversary has to capture almost 1300 sensor nodes. It indicates that the resilience ability of the proposed PPBR scheme is better than that of the other three schemes. The reason is that in this scheme if the adversary wants to capture a normal communication link, it must crack the *t* + 1 coefficients of the polynomial and decrypt the shared keys among the sensor nodes simultaneously. Consequently, it is more difficult than the compared schemes above. Comparing Figure 10 and Figure 11, with the decrease in direct connectivity, for example, when *p* is reduced from 0.5 to 0.33, the number of permitted captured nodes increases obviously. When the number of captured nodes is greater than a certain threshold, the capture probability of normal nodes will be pretty high in the E-G scheme, Liu scheme and Amar scheme. The whole network could be in danger as the ratio of the number of captured nodes and the total number of nodes of the whole network exceeds a certain value.

In this paper, sensor nodes can directly or indirectly build a link to a MS. After a round of data collection, sensor nodes can find an appropriate tree-based path to establish communication links with the MS no matter what changes in the topology occur, including nodes’ death, the addition of new nodes, or dynamic changes in the intermediate nodes. For a new round of data collection, the sensor nodes will also follow the steps in the scheme above.

## 5. Conclusions

This article puts forward a hybrid key management scheme for wireless sensor networks with MS based on a polynomial pool and basic random key pre-distribution. A tree-based key discovery strategy was introduced in the path key establishment phase. The proposed scheme takes full advantages of these two kinds of methods, and comprehensively considers various performances of the system. It can make full use of the heterogeneity between the ordinary sensor nodes and MS to save the storage space of the ordinary sensor nodes by adequately increasing the storage utilization rate of the MS on the premise of satisfying a certain connectivity. In terms of connectivity, it can make the sensor nodes link with the MS as much as possible via the tree-based path key discovery phase, thus improving the connectivity of the whole network. On the premise of solving the problem of the *t*-degree property of the polynomial, the proposed scheme can make it more difficult for an adversary to capture the sensor nodes by means of integrating the basic random key predistribution scheme, and it improves the resilience of the sensor network. In most practical application scenarios, sometimes the sensor nodes are not absolutely fixed, they can move with some certain velocities. In future work, we will further consider the complex mobile network model. It means we need to further extend this scheme to the networks with mobile sensor nodes, not only mobile sink nodes. In that case, we need to evaluate the influence on key management of topology changes due to the relative motion among the sensor nodes, and we need to investigate a secure handover mechanism when the communication links are disconnected due to the sensor node movement. Furthermore, we will verify the specific performances of the scheme in a practical platform.
